# The Unfavorable Alliance of Pain and Poor Sleep in Children with Life-Limiting Conditions and Severe Psychomotor Impairment

**DOI:** 10.3390/children5070082

**Published:** 2018-06-21

**Authors:** Larissa Alice Dreier, Julia Wager, Markus Blankenburg, Boris Zernikow

**Affiliations:** 1 Paediatric Palliative Care Centre, Children’s and Adolescents’ Hospital, 45711 Datteln, Germany; l.dreier@kinderpalliativzentrum.de (L.A.D.); b.zernikow@deutsches-kinderschmerzzentrum.de (B.Z.); 2 Department of Children’s Pain Therapy and Paediatric Palliative Care, Faculty of Health, School of Medicine, Witten/Herdecke University, 58448 Witten, Germany; 3 Paediatric Neurology, Psychosomatics and Pain Therapy, Center for Child, Youth and Women’s Health, Klinikum Stuttgart, Olgahospital/Frauenklinik, 70174 Stuttgart, Germany; m.blankenburg@klinikum-stuttgart.de

**Keywords:** sleep, pain, children, psychomotor, impairment, neurological

## Abstract

A high prevalence of sleep problems exists in children and adolescents with life-limiting conditions (LLC) and severe psychomotor impairment (SPMI). This study aimed to compare the impacts of various child-related (pain, epilepsy, repositioning, medical care) and environment-related (light, noise, TV/radio, open door) factors on sleep in this vulnerable population. Data were obtained through the “Sleep Questionnaire for Children with Severe Psychomotor Impairment” (SNAKE) by proxy assessment. *n* = 212 children (mean age: 10.4 years) were included in the analyses. Logistic and linear regression models were used to compare child- and environment-related factors against the global rating of children’s sleep quality, five SNAKE scales, children’s sleep duration, and sleep efficacy. Pain increased the risk of sleeping poorly four-fold (OR (odds ratio) = 4.13; 95% CI (confidence interval): 1.87–9.13) and predicted four sleep problems as assessed by the SNAKE. Children who needed to reposition during the night were at three times greater risk of sleeping poorly (OR = 3.08; 95% CI: 1.42–6.69). Three of the five SNAKE scales were predicted through nocturnal repositioning. Repositioning and epilepsy predicted a reduced sleep duration and low sleep efficacy. None of the environment-related factors exhibited statistically significant results. This study emphasizes the urgent need for reliable pain detection in the context of sleep disturbances in severely ill children.

## 1. Introduction

Life-limiting conditions (LLC) include a large variety of illnesses from which affected children and adolescents probably die before reaching adulthood [1,2,3]. The majority of affected children experience severe psychomotor impairments (SPMI), meaning that they are impacted by both physical disabilities and intellectual impairment [4,5]. In pediatric palliative care, not malignant, but rather genetic (e.g., trisomy disorders), metabolic (e.g., mucopolysaccharidosis, cystic fibrosis) and neurologic diseases (e.g., West syndrome, epileptic encephalopathy) constitute the most common diagnoses [6,7,8]. These diagnoses are accompanied by numerous complex, simultaneously occurring, and often distressing symptoms [9]. With a prevalence rate of between 50–80%, sleep disturbances are one common symptom that is very exhausting for patients and their parents [3,5,10,11].

To date, the etiology of sleep problems in children with LLC and SPMI is not entirely understood [5]. Comprehensive information on possible influencing factors would be beneficial for clinical practice and help to reduce patients’ and families’ suffering [5]. To address this, several studies have identified influencing factors that could negatively impact children’s sleep [4,12]. Most studies have investigated factors that are child-related, such as epilepsy [13,14,15], repositioning [16,17,18], medical care [19,20], and pain [21,22,23].

In one of the more comprehensive studies, Hemmingson et al. [24] analyzed the association between individual child-related factors and sleep problems in disabled children and identified that the presence of pain was the strongest contributing factor. However, the authors did not use a validated sleep measure and only included children with motor disabilities [24]. As a consequence, the results are only transferable to children with LLC to a limited extent.

Another important aspect that is likely to negatively impact sleep in children is the sleep environment [25,26]. There are a small but growing number of studies assessing the impact of environment-related factors, such as noise or light, on the sleep of children with SPMI [27,28]. The sleep of children with multiple disabilities may be impeded when their bedroom is not completely dark or when it is too hot or too cold [29]. Parents are fundamental figures to promote a supportive sleep environment [30]. For instance, parents of children with Sanfilipo syndrome reported that they had employed behavioral modifications to promote sleep, including an individual bedroom for their child, solid and secure doors throughout the house, and closed-circuit television [31]. Existing evidence recommends environmental influences should be considered when diagnosing sleep problems in children with neurological impairments; however, the direct influence of individual environment-related factors on sleep in this population has not been well-studied and thus may be difficult to assess [11,32,33]. Additionally, due to the heterogeneous clinical pictures and diagnoses in this population, findings from currently available literature cannot be generalized for children with SPMI.

The current study intends to address the paucity of scientific literature investigating the impact of both child-related and environment-related factors on severely disabled children’s sleep.

The aim of this study is to compare the influence of child-related and environment-related factors on sleep in children with different LLC and SPMI based on reports by their parents on the validated “Sleep Questionnaire for Children with Severe Psychomotor Impairment” (SNAKE). As the majority of children with SPMI have speech impairment and hence are unable to report the causes of their sleep disturbances independently, the information gathered from parents is particularly important as it contributes to a clearer understanding of circumstances that might promote sleep problems in children with SPMI [6,7].

## 2. Materials and Methods

### 2.1. Dataset

The analyses reported in this study were based on a comprehensive dataset of *n* = 226 children and adolescents (with an age range of 1–25 years) with SPMI [4]. Inclusion criteria for initial recruiting were defined as follows:

- Child is affected by intellectual disability and at least one additional physical disability;
- Parents/caregivers are able to adequately communicate in German;
- Patient is being cared for in one of the participating institutions;
- Parents consented to participation and the use of the patient’s data for study purposes [4].

Specific diagnoses were not an entry requirement, and as a result children participating in this research represented the heterogeneity of illnesses children with LLC and SPMI [4]. Children and their parents or caregivers were originally recruited from three inpatient institutions (children’s hospital, children’s hospice, respite care) and one outpatient facility (pediatric palliative home care) and Germany Ethical approval (Ethics Committee of the Children’s and Adolescents’ Hospital Datteln, Germany; Approval code 2008/06/26/MB) and informed consent were obtained. The data from each patient were based on a proxy report of parents or caregivers assessed through the SNAKE [4]. More detailed information on children’s recruitment can be found in studies based on the same dataset but with different questions that have been published previously [4,34,35,36].

### 2.2. Sleep Questionnaire for Children with Severe Psychomotor Impairment (SNAKE)

The SNAKE is specially designed to assess sleep in children with SPMI and is based on proxy reporting by parents or caregivers [4]. It is a reliable and valid questionnaire that is stable over time, applicable to a broad age group (1–25 years), and addresses a wide spectrum of sleep problems based on the International Classification of Sleep Disorders (ICSD-2; [4,37]). The SNAKE consists of 54 items and regards a child’s sleep during the past four weeks within five scales (disturbances going to sleep, disturbances remaining asleep, arousal and breathing disorders, daytime sleepiness, and daytime behavior disorders). Additionally, the SNAKE assesses suspected risk factors of sleep problems (e.g., pain, repositioning, epileptic seizures, medical care, open door in child’s bedroom, noise in child’s bedroom, light in child’s bedroom, TV running in child’s bedroom) and provides a global rating of a child’s sleep quality, sleep duration, and sleep efficacy as well as the general core characteristics (child’s sex, age, diagnosis or diagnoses, height, weight, medication(s), the parents’ marital status, where the child predominantly lives, the number of children in the household, the number of people in the household, who filled out the questionnaire) of the child and family that do not fit into one of the five scales [4].

The overall ratings of a child’s sleep during the past four weeks were dichotomized into “good sleep” (“very good” and “good”) and “poor sleep” (“very poor”, “poor” and “satisfactory”). Sleep efficacy equals the quotient between a child’s sleep duration (“How many hours of sleep did your child actually get at night?”) and the time he or she has spent in bed (“At what time did your child usually wake up in the morning” minus “At what time did you usually put your child to bed at night?”) multiplied by 100 (specification in percent) [4].

### 2.3. Child- and Environment-Related Factors

The variables grouped into child-related factors (CRF) were pain, epileptic seizures, repositioning, and medical care during the night. Environment-related factors (ERF) included an open door in the child’s bedroom during the night, light in the child’s bedroom, TV/radio running in the child’s bedroom, and noise in the child’s bedroom. For these items, parents or caregivers were asked how often a particular factor was present during the past four weeks. Answers were scored on a four-point scale (“three or more times a week”, “once or twice a week”, “less than once a week”, “never”). For regression analyses, the CRF and ERF were recoded and dichotomized as follows: “never” into “no” (factor not present during the past four weeks), “less than once a week”, “once or twice a week” and “three or more times a week” into “yes” (factor present during the past four weeks).

### 2.4. Statistical Analyses

Data were analyzed using SPSS (IBM, version 25). The general characteristics of the patients were assessed through descriptive statistics. A multiple logistic regression was conducted to relate the presence of the individual CRF and ERF (dichotomous variable) to the global rating of a child’s sleep. “Good sleep” during the past four weeks was set as the reference category. Multiple linear regression analyses were used to analyze the influence of the individual CRF and ERF on the five scales of SNAKE, as well as a child’s sleep duration and sleep efficacy. For all regression analyses, the enter method was used to find the final model. The level of significance was defined as *p* = 0.05.

## 3. Results

### 3.1. Sample Characteristics

Of the *n* = 226 children contained in the dataset, *n* = 212 children (mean age: 10.4, SD = 5.52; *n* = 99 (46.7%) female, *n* = 113 (53.3%) male) were eligible for analyses. *n* = 185 (88.1%) children were under 18 years of age, *n* = 25 (11.9%) were above 18 years of age. Fourteen children had to be excluded due to missing values (more than 50% missing) in the SNAKE (*n* = 2) or the core parameters (*n* = 12). Regarding the three inpatient institutions, most children were recruited from the children’s hospital (*n =* 130; 61.3%, respite care: *n* = 43; 20.3%, children’s hospice: *n* = 15; 7.1%). From the outpatient facility, *n* = 24 children (11.3%) were recruited. The most common diagnoses of *n* = 212 children were cerebral palsy (*n* = 54; 26.3%), global developmental retardation of unknown origin (*n* = 35; 17.1%), different rare syndromes (*n* = 28; 13.7%), and metabolic disorder/neurodegenerative disease (*n* = 21; 10.2%). Regarding consecutive symptoms of the underlying illnesses, *n* = 110 children (55.8%) had epilepsy, *n* = 28 children (14.5%) were visually impaired, and *n* = 6 (3.7%) children experienced total deafness. The ability to hear was impaired or supported by suitable tools in *n* = 25 children (15.2%).

One hundred sixty-two (84.4%) children received medications. Of these, *n* = 117 (61.6%) children were treated with anticonvulsant medications and only *n* = 19 (9.6%) received analgesics. For an exact listing of analgesics, see Table 1.

### 3.2. Frequency of CRF and ERF

Approximately 60.8% of parents reported the presence of at least one CRF (one factor present: 23.1%; two factors present: 24.1%; three factors present: 11.8%; four factors present: 1.9%) and 73.6% reported at least one ERF (one factor present: 39.2%; two factors present: 22.2%; three factors present: 10.4%; four factors present: 1.9%).

**Figure 1:**
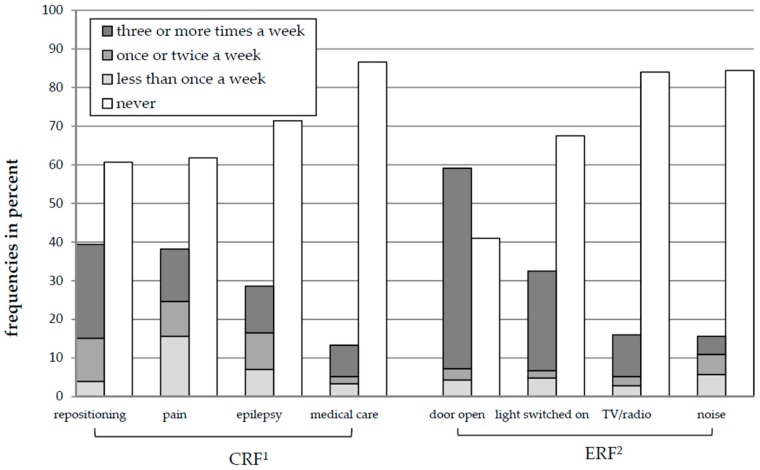
Frequencies of child-related factors (CRF) and environment-related factors (ERF). ^1^ answers the question, “How often did your child have a poor sleep because of…”; ^2^ answers the question, “How often was the occurrence of…”.

A closer look at the single CRF shows that the majority of parents did not consider a single CRF relevant for sleep interference of the child during the last four weeks (no interference via repositioning: 60.7%; pain: 61.8%; epilepsy: 71.4%; medical care: 86.6%; see Figure 1). For ERF, a similar pattern was evident; many parents reported that the light in the child’s bedroom was not switched on (67.5%), no TV/radio was running in the child’s bedroom (84%), and that noise was not present in the child’s bedroom (84.4%). Contrary to the other factors, the majority of parents reported that the door in child’s bedroom was open “three or more times a week” (51.9%; Figure 1).

### 3.3. Influence of CRF and ERF on the Global Ratings of Children’s Sleep

Multiple logistic regression analysis was performed to determine the relationship between CRF and ERF and the global rating of a child’s sleep. The response categories of all variables were dichotomized.

*n* = 42 (19.8%) children were rated as having a very good, *n* = 67 (31.6%) good, *n* = 70 (33%) satisfactory, *n* = 26 (12.3%) poor, and *n* = 7 (3.3%) very poor sleep during the past four weeks (global sleep rating). Children were nearly equally allocated to the two dichotomized groups (good sleep: *n* = 109, 51.4%; poor sleep: *n* = 103, 48.6%).

**Figure 2:**
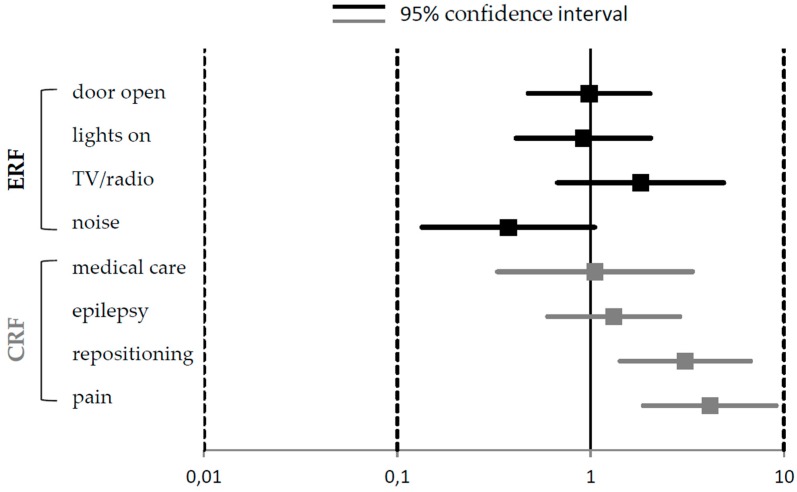
Odds Ratio (OR; with 95% confidence interval) of multiple logistic regression between individual CRF and ERF and the global sleep rating.

Analyses revealed that children who had pain during the past four weeks were over four times more likely to have bad sleep (odds ratio; OR = 4.13 (95% confidence interval; CI: 1.87–9.13)). The risk of sleeping badly was three times greater (OR: 3.08 (95% CI: 1.42–6.69)) when a child needed to be repositioned during the night. None of the other CRF or ERF was identified as significant influencing factors (Figure 2).

### 3.4. Influence of CRF and ERF on the SNAKE Scales

Table 2 demonstrates the influence of CRF and ERF on specific pediatric sleep problems obtained according to the five scales of the SNAKE. Multiple linear regression analyses revealed that in comparison with other CRF and ERF, pain predicted the most sleep problems (disturbances going to sleep: β = 0.36, *p* < 0.001; disturbances remaining asleep: β = 0.28, *p* < 0.001; arousal and breathing disorders: β = 0.26, *p* < 0.01; daytime behavior disorders: β = 0.23, *p* < 0.01). No significant relation was found between having pain and daytime sleepiness. By contrast, epilepsy constituted the strongest predictor for daytime sleepiness (β = 0.37, *p* < 0.001). Significant relationships were found between repositioning and three of the five SNAKE scales (disturbances remaining asleep: β = 0.22, *p* < 0.01; arousal and breathing disorders: β = 0.29, *p* < 0.001; daytime sleepiness: β = 0.16, *p* < 0.05). Medical care and a single ERF had no impact on the five SNAKE scales (all *p* > 0.05).

### 3.5. Influence of CRF and ERF on Sleep Duration and Sleep Efficacy

Linear regression models tested whether there was a significant relation between CRF, ERF and sleep duration and sleep efficacy of children with SPMI.

As depicted in Table 2, repositioning constituted a statistically significant negative predictor for sleep duration (β = −0.18, *p* < 0.05) and sleep efficacy (β = −0.21, *p* < 0.05). Further, having epilepsy predicted a significantly lower sleep efficacy (β = −0.22, *p* < 0.05). No other CRF and none of the ERF were related to sleep duration or sleep efficacy (all *p* > 0.05).

## 4. Discussion

The present study investigated possible influencing factors on sleep in children with SPMI. This topic is particularly important because of the high prevalence rate of sleep problems in this vulnerable population [5]. The results revealed that having pain was the most predictive factor for sleep problems in these children. Environment-related factors were not associated with children’s sleep.

### 4.1. Child-Related Factors and their Influence on Sleep

Children whose caregivers stated that their child suffered from pain during the past four weeks had a four-fold increased risk of sleeping poorly. This key finding constitutes additional proof that pain is associated with sleep problems in children with severe disabilities [21,39,40]. Moreover, this finding is in line with the findings of Hemmingsson et al. [24], who identified having pain as the most predictive factor of sleep problems in children with multiple disabilities. The magnitude of the effect reported by Hemmingsson et al. (OR = 3.4) is comparable to the results of the current study.

With the exception of daytime sleepiness, having pain additionally predicted worse functioning in four of the five scales of the SNAKE (disturbances going to sleep, disturbances remaining asleep, arousal and breathing disorders, daytime behavior disorders). Unfortunately, although a number of studies assume a relationship between pain and sleep in children with severe disabilities [21,39,40], approaches that directly compare pain and specific sleep problems are scarce. One existing study of Breau and Camfield [21] indicated a relationship between pain and nocturnal waking as well as between pain and sleep disordered breathing in disabled children, which is concordant with our results [21].

The lack of a relationship between pain and increased daytime sleepiness is interesting because one would expect that daytime functioning is likewise affected if a child’s night-time sleep is disturbed through pain. There are several studies that suggest that poor night-time sleep negatively affects children’s daytime behavior [4,10,24]. In contrast, the influence of pain on daytime sleepiness is still insufficiently explored. It is plausible that children included in this study experienced pain present during both the night-time and daytime [23,41]. Pain during the day may interrupt daytime sleepiness, even though the child is tired.

Contrary to the findings relating to pain, repositioning and epilepsy were both significant predictors for daytime sleepiness. Immobile pediatric patients need to be repositioned regularly to prevent pressure ulcers [42,43,44]. Repositioning is a care measure carried out by caregivers to increase a patient’s comfort [45]. Unlike pain, it underlies, to some extent, the caregiver’s control, is time-limited, and does not disturb a patient’s sleep unexpectedly. Therefore, it is plausible that if a child suffers from poor night-time sleep due to nocturnal repositioning, he or she might be sleepy during the daytime [46].

In those with epilepsy, it is highly plausible that children may be woken several times due to nocturnal seizures, thus increasing daytime fatigue, an assumption premise that is supported by several studies [18,47,48,49]. We found that epilepsy is a significant factor for sleep disturbances and reduced sleep efficacy, which emphasizes epilepsy’s potential for disrupting children’s sleep. Beyond this, it is possible that children who received PRN (pro re nata; as needed) medication during nocturnal seizures may be sleepy during the daytime because of the sustained drug effect.

The need for repositioning during the night yielded a three times greater risk of poor sleep in general and therefore, after pain, constitutes the second most influential factor in our sample. Repositioning also negatively influenced sleep duration and sleep efficacy. In accordance with our results, Bloetzer et al. [17] found that repositioning increased the risk of having a sleep disorder by seven-fold.

Our analyses identified few associations between the ERF and CRF investigated in this study and daytime behavior disorders. Didden et al. [50] found that sleep problems in children with intellectual disabilities promoted daytime problem behaviors, such as aggression, hyperactivity, and non-compliance. It is possible that we did not find such a relationship because severely disabled children are affected by a variety of complex factors (e.g., primary disease, medication) that are not considered in these analyses. Nevertheless, the patient’s level of intellectual disability in the cited study ranged from mild to severe and was not necessarily accompanied by psychomotor impairment [50], while children in our study were impacted not only by intellectual, but severe intellectual and physical impairment. For this reason, transferring results from this study to our population is only feasible to a limited extent and should be done with caution.

Beyond repositioning, medical care during the night did not influence the assessed sleep variables. This may be somewhat surprising as one would expect medical care to interrupt the child’s sleep. Similar results, however, have been reported for the use of postural devices, such as night orthoses in children with cerebral palsy [20,51] and ventilation [52]. One explanation is that the term “medical care” is rather unspecific and might be interpreted differently by various caregivers. Future work should consider dividing this term into specific medical measures to gain more specific information.

### 4.2. Environment-Related Factors and their Influence on Sleep

Another key finding is that no significant impact of any single ERF was found on the global rating of children’s sleep on the five SNAKE scales or on sleep duration and sleep efficacy. Analyzing this topic was important because some studies recommend attending to environmental factors when attempting to improve disabled children’s sleep [10,11,27,28]. The question of how factors such as light, noise, or open doors severely affect disabled children’s sleep problems remains unanswered.

Only a small number of children in this study suffered from visual impairment. The non-existent relationship between ambient light and children’s sleep problems can therefore not be traced to the fact that children were just not able to perceive light. A similar assertion can be made for factors such as ambient noise or TV/radio, which presuppose an unimpaired hearing ability. Here too, the majority of the study sample was able to hear, which leads to a similar conclusion as mentioned above.

Even if our results could be interpreted in the sense that environmental factors seem to be irrelevant for global and specific sleep problems in a population of children with SPMI, one cannot easily draw such a conclusion. Other factors that were not controlled in the scope of the study (e.g., parental bias or interpretation when answering the SNAKE, intentional avoidance of factors that might be adverse for child) may be playing a role in the non-significant findings reported here. The data indicate that only a small number of patients were exposed to light, noise, or TV/radio sounds, which may result in a lack of thorough assessment of the variables and low statistical power. Further research is needed to investigate whether environment-related factors are indeed less relevant for disabled children’s sleep.

### 4.3. Clinical Implications

This paper contains important information for clinicians in pediatric palliative care. When evaluating possible reasons for sleep problems in the vulnerable population of children and adolescents with LLC and SPMI, special attention should be paid to signs of pain. For evaluating those signs, parental descriptions can be helpful [53,54]. Epilepsy and the need for repositioning both influenced children’s night-time sleep negatively, according to parental reports. Even if these two factors cannot be fully eliminated, clinicians should regularly review the patient’s medication doses to potentially reduce the frequency or duration of seizures during the night, as well as consider the impact of medications on daytime sleepiness. Further, it should be determined whether the frequency of repositioning a child is appropriate or could possibly be reduced to minimize the number of nocturnal awakenings. Our results imply that ERF seem to be less relevant than CRF for disabled children’s sleep, which might indicate that clinicians should focus on CRF when assessing and treating sleep problems in this population. Considering the fact that we cannot definitively state whether the assessed ERF were irrelevant for children’s sleep problems or avoided by parents, an assertion such as this should be drawn with caution and requires future research.

### 4.4. Limitations

A number of limitations were present in our study. Firstly, some highly relevant data on characteristics of the children and families included in this study (e.g., frequency of repositioning during the night, timing of drug administration) were not collected. These limited interpretations of the findings should be considered in future studies to obtain a deeper comprehension of influencing factors for disabled children’s sleep. Secondly, we did not assess the impact of, or statistically control for, some key participant demographics, such as primary disease or age. This is the first study to investigate a large number of child-related and environment-related factors that may impact sleep in children with severe disabilities, and future studies with larger sample sizes should consider furthering these findings by incorporating these additional, potentially influential factors. Because children with SPMI differ extremely in their developmental stages and cannot easily be compared following their chronological age, determining reasonable cut-off points for assessing different age groups is difficult. Nevertheless, finding these cut-off points would be an interesting and important aspect for future research efforts.

Thirdly, our results are solely based on the caregiver’s appraisal and thus are highly subjective. Objective measures, such as polysomnography or actigraphy, would be helpful as a second approach to investigate our line of questioning. Another limitation is that the SNAKE does not contain cut-off values that define when a particular sleep problem is clinically conspicuous. For the present paper, this point is acceptable because our aim was to obtain an understanding of relationships between sleep problems and potentially influencing factors. However, it would be helpful for clinicians and subsequent studies to define cut-off values for the SNAKE to better identify individuals who may be impacted by CRF and ERF, which can then be modified for improved sleep.

Finally, the data in this study originated from an existing dataset and were not collected prospectively, which constitutes another limitation.

## 5. Conclusions

The relationship between several CRF and ERF and sleep in children with LLI and SPMI were assessed. The presence of pain was identified as the most influential factor for sleep problems of severely disabled children, followed by the need for repositioning. Clinicians should consider this result when deciding on treatment strategies for these children. By contrast, in this study none of the ERF had an impact on severely disabled children’s sleep problems. This result may imply that future studies should focus first on CRF rather than ERF when attempting to attain a deeper understanding of causes of sleep problems in severely disabled children. Subsequent studies should extend knowledge on the examined factors and include patient and family characteristics that were not included in this study.

## Figures and Tables

**Table 1:** Listing of received analgesics.

Drug Class ^1^	Active Agent	N
Non-opioid analgesics	Dipyrone	8
	Ibuprofen	2
	Acetylsalicylic acid	2
	Paracetamol	2
Weak opioids	Tilidine	2
Strong opioids	Morphine	4
	Opium tincture	1
	Oxycodone	1
Cannabinoids	Delta-9-Tetrahydrocannabinol	1
		23

^1^ Classification derived from the WHO (World Health Organization) analgesic ladder [38].

**Table 2:** Linear regression analyses with CRF and ERF as independent variables and “Sleep Questionnaire for Children with Severe Psychomotor Impairment” (SNAKE) scales, sleep duration, and sleep efficacy as dependent variables.

		**Dependant Variables: SNAKE Scales 1–5**														
		**Scale 1** **(Disturbances Going to Sleep)**			**Scale 2** **(Disturbances Remaining Asleep)**			**Scale 3** **(Arousal and Breathing Disorders)**			**Scale 4** **(Daytime Sleepiness)**			**Scale 5** **(Daytime Behaviour Disorders)**		
	**Variable**	**B (SE)**	**β**	**t**	**B (SE)**	**β**	**t**	**B (SE)**	**β**	**t**	**B (SE)**	**β**	**t**	**B (SE)**	**β**	**t**
CRF	Pain	3.10 (0.71)	0.36	4.33 ***	2.76 (0.74)	0.28	3.70 ***	2.43 (0.69)	0.26	3.51 **	0.24 (0.47)	0.04	0.50	1.76 (0.66)	0.23	2.66 **
	Repositioning	0.12 (0.69)	0.01	0.17	2.16 (0.72)	0.22	2.98 **	2.66 (0.67)	0.29	3.95 ***	0.98 (0.46)	0.16	2.13 *	0.08 (0.64)	0.01	0.13
	Epilepsy	−0.81 (0.69)	−0.08	−1.17	1.70 (0.72)	0.16	2.34 *	0.77 (0.67)	0.07	1.15	2.36 (0.46)	0.37	5.13 ***	0.59 (0.65)	0.07	0.90
	Medical care	0.86 (1.02)	0.06	0.84	−0.03 (1.06)	−0.00	−0.03	0.51 (0.98)	0.03	0.52	−0.34 (0.67)	−0.03	−0.50	−0.00 (0.93)	−0.00	−0.00
ERF	Noise	−0.16 (0.82)	−0.01	−0.19	−0.16 (0.85)	−0.01	−0.18	1.10 (0.79)	0.09	1.38	0.51 (0.54)	0.06	0.94	0.98 (0.75)	0.10	1.30
	TV/Radio	−0.19 (0.85)	−0.01	−0.23	0.83 (0.88)	0.06	0.94	−1.09 (0.82)	−0.08	−1.32	23 (0.56)	0.02	0.41	0.13 (0.78)	0.01	0.17
	Lights on	0.58 (0.68)	0.06	0.84	0.90 (0.71)	0.09	1.27	0.47 (0.66)	0.05	0.71	0.76 (0.45)	0.12	1.66	0.01 (0.63)	0.00	0.02
	Door open	0.95 (0.61)	0.11	1.54	0.23 (0.64)	0.02	0.36	0.22 (0.59)	0.02	0.37	−0.13 (0.40)	−0.02	−0.33	0.15 (0.57)	0.02	0.26
		**Dependant Variables: Sleep Duration and Sleep Efficacy**														
		**Sleep Duration**			**Sleep Efficacy**											
	**Variable**	**B (SE)**	**β**	**t**	**B (SE)**	**β**	**t**									
CRF	Pain	−0.56 (0.35)	−0.14	−1.58	−3.98 (3.25)	−0.11	−1.22									
	Repositioning	−0.74 (0.34)	−0.18	−2.15 *	−7.49 (3.19)	−0.21	−2.34 *									
	Epilepsy	−0.38 (0.34)	−0.08	−1.11	−8.53 (3.14)	−0.22	−2.71 *									
	Medical care	0.66 (0.50)	0.10	1.31	5.25 (4.40)	0.10	1.19									
ERF	Noise	0.40 (0.40)	0.07	1.01	5.35 (3.51)	0.12	1.52									
	TV/Radio	−0.32 (0.41)	−0.06	−0.78	−4.29 (3.78)	−0.09	−1.13									
	Lights on	−0.36 (0.33)	−0.08	−1.07	−2.89 (3.05)	−0.08	−0.94									
	Door open	−0.16 (0.30)	−0.04	−0.54	1.82 (2.74)	0.05	0.66									

CRF: child-related factors, ERF: environment-related factors; B: regression coefficient, SE: standard error, β: beta, t: T- statistics; *p < 0.05; ** *p* < 0.01; *** *p* < 0.001.
